# Some Observations on the Carlsbad Treatment

**Published:** 1881-07

**Authors:** E. T. Bruen


### Selections.

### SOME OBSERVATIONS ON THE CARLSBAD TREATMENT.

By E. T. BRUEN, M.D.

Read before the Philadelphia County Society, January 26, 1881.

In common with many of those I address this evening, I have used with much good effect in selected cases the Carlsbad salt. It is needful only to remind this body of the more powerful physiological action of certain drugs when administered in dilute solution, to explain the advantages of these prepared salts. One of the most original applications of the above facts to the treatment of disease will be found in a paper by Dr. John Guiteras in the *Philadelphia Medical Times* for June 5, 1880, entitled "The Therapeutic Advantages of Administering the Iodide of Potassium Fasting, with Some Remarks on Interstitial Hepatitis with Enlargement of the Liver." In this paper is discussed the advantage of administering alternative drugs in dilute solution while fasting, and the opinion is expressed that the physiological action of the drug is much intensified. I can confidently corroborate the statements of Dr. Guiteras, and as, during a recent visit to Europe, I spent some time at a number of Baths, including Carlsbad, I will offer the results of my notes: these comprise the varieties of cases I found in the habit of visiting Carlsbad, the benefit derived, the action of the waters, and the after-treatment advised by the local physicians. It must be borne in mind that the Carlsbad salts do not represent the waters as they issue from the laboratory of nature. The Carlsbad salt is merely, after all, Glauber's salt: the true waters contain, in addition, carbonate of sodium 13 grains, sulphate of sodium 20 grains, to the pint, besides a fair amount of chloride of sodium, some carbonate of lime and magnesium, with free carbonic acid, at a varying temperature from 122° Fahr. to 166° Fahr. I am inclined to think that those who consult the books published by the resident physicians at the German baths will find them to yield information comparable to the Yankee's definition of a flea, viz., "A critter which, when you put your finger on it, warn't there." However, in a recent book, "Carlsbad: Its Natural Healing Agents," by Dr. J. Kraus the reader will find a good analysis of the waters, some facts as to the climatology of the place, and, included in a letter from an ex-patient, many useful hints are given as to the method of life, the dietary most suitable, the personal hygiene, and the best route to follow in a journey to Carlsbad.

The cases resorting to Carlsbad may be divided into three classes: 1. Cases of enlargement of the liver and spleen, as a sequence to repeated congestions induced by continued dyspepsia or chronic malaria, interstitial hepatitis, or primary stage of cirrhosis, especially when jaundice and deficient intestinal digestion persist, and also cases of chronic indigestion with deficient assimilation, whether or not constipation be a prominent symptom. 2. Cases of chronic rheumatism or gout. 3. Cases of the gouty state, or those obscure cases attended with renal congestion or inactivity, as evidenced by the passage of a deficient amount of urine of low specific gravity, usually associated with deficient vaso-motor tonus. These cases, as I shall show later, are often much benefited, some cured, by a course of the waters. The springs differ from each other chiefly in temperature, which is high where it issues from the source, ranging from 166° Fahr. to 122° Fahr., and in the amount of carbonic acid contained in them. It is usual for patients to rise about six o'clock and to spend about two hours at the spring, taking at fifteen minutes, interval three or four ounces of water. Beginners usually indulge in from twelve to sixteen ounces a day; the amount is often carried up to twenty-four or thirty ounces. Exercise is taken while drinking the waters, and then resort is had to the hotel. Later I shall observe that a strict diet is fundamental to the success of "the cure:" the diet consists of a light breakfast of eggs, bread, and coffee, at noon, meat (steak or chicken) constitutes the meal; in the evening the same meal is repeated. No one under the "cure"

will venture on a *table-d'hote*, or even a more liberal meal, but this strict regimen is possible, since every one eats at one of the numerous restaurants, one of the features of the place. Early hours and moderate exercise are especially enjoined. Now as to the effects produced. Most persons experience a laxative action, not a purgative effect; but I have known other cases in which the compound liquorice powder was used daily to produce movements of the bowels. Without exception, individuals experience the most profound exhaustion, and usually profound anæmia ensues. In most cases the urine is notably increasd in amount, and at times is of a blackish green color. The stools are often greenish, doubtless owing to the increased secretion of bile; in the urine the color is perhaps due to the destruction of the red blood-corpuscles. For it would seem that the alterative effect of the waters is so great as to make the systemic condition resemble that induced by too generous use of the potassium salts.

Patients have frequently said to me, "Doctor, I cannot endure this treatment; it is too reducing; my strength is ebbing away." Notwithstanding this, the treatment is continued three weeks or a month, the period of the "cure," and the patient is dispatched to Ischl, St. Moritz or some other springs, the waters of which contain iron, and thus the blood crasis is restored. In persons weakened by previous long sickness, recuperation is very slow after a Carlsbad course; indeed it appeared to me that the treatment is pushed too far with these cases. Sir Henry Thompson has said that he is quite well satisfied with a smaller amount, "say six to eight ounces given daily during six or nine weeks, instead of the usual three weeks of the foreign course." I incline to think that the restricted diet contributes very much to the favorable result usually obtained.

To illustrate: alcohol, or any fermented liquor, is to be used in its most dilute and purest form, or relinquished altogether; sugar, fatty matters, butter, cream, fat of meat, are proscribed; the fruits are not allowed, and vegetables and good fish are not attainable. In serious cases, a repetition of the course every three or four months is found advantageous, provided the patient's strength at all permit; too much must not be hoped for from a single course. The effect of the salts of potash, when administered continuously, is to increase the oxidation of the tissues, and anæmia is brought about. Apparently the same effect can be brought about by the salts contained in these waters.

Let me now append the outline of the history of two cases which I had the opportunity to examine in Carlsbad, both before and after treatment:

A gentleman, a resident of one of our Southern States, had been subject to frequent attacks of chills and fever, had been through an attack of yellow fever, and had previously been broken down by life in the Southern army during our late war. The liver extended three fingerbreadths below the ribs, the spleen included three times its normal area, there was continued pain over the liver, with intestinal indigestion, hemorrhoids, and constipation. After a three-weeks' course, the liver was reduced to normal size, the spleen also, but the exhaustion was extreme, there had been a loss of nearly twenty pounds of flesh, anæmia was profound, digestion was capricious, the hemorrhoidal veins dilated. In all the patient's stay at Carlsbad no more than a laxative effect had been produced by the waters. In this case, after eight weeks' stay at Ischl and St. Moritz, a general improvement occurred, and when I left him in Paris in September he was better than he had been for years, and the anæmia was yielding to small doses of iron and bitter tonics.

The second case was that of a gentleman from New York, fifty years of age, in whom the liver and spleen were also much enlarged, and dyspepsia and anæmia very pronounced. In this case, also, reduction in the size of both liver and spleen occurred, but the force of the cardiac beat was alarmingly reduced. There were attacks of fainting, with fluttering action of the heart; also numbness of the arms and legs was freqnently complained of, and the temptation was strong to discontinue the treatment; but it was persevered in for four weeks. In this case recuperation has been very slow, and at date the strength of this gentleman is less than before the treatment. At the same time the appetite is good, and the anæmia is slowly abating. At the termination of the Carlsbad course the springs of Franzenbad were selected for this patient, because of their contiguity to Carlsbad. The patient's weakness after the course was so extreme that a long journey was impossible.

The waters of these springs are ferruginous, and, as I shall presently state, it is the custom to follow the Carlsbad course with a tonic regimen. The cases exhibit how powerful a therapeutic ally we possess in the Carlsbad waters; but the cases must be carefully managed while under treatment.

I noticed a group of cases at Carlsbad sent there for the mitigation of symptoms, such as come-and-go headache, ascribed to vaso motor weakness, evidenced by the tendency to change color readily. In these cases there was often associated flatulent dyspepsia. The stimulation of the carbonic acid in the waters seemed to be beneficial by equalizing the circulation: the headache would disappear, the tongue become clean, and the dyspepsia often vanished. Some English gentlemen told me they could only secure a clean tongue by an occasional visit to Carlsbad. The headache I have designated as come-and-go headache because it is peculiar in this respect, that it is not persistent, but comes on suddenly in the night or at any part of the day. It is temporarily relieved by any warm not too stimulating drink, such as these waters. I noticed, however, that warm milk frequently seemed of as much service; but the patients themselves were satisfied that Carlsbad was alone their resource.

A word as to the climate of Carlsbad. This is frequently variable, as in all high latitudes, and an abundant supply of clothing of different textures is advisable.

The after-cure consists in dispatching the patient to some mountainous resort possessed of a ferruginous spring. At present two localities are fashionable—Ischl, in the Austrian Tyrol, and St. Moritz, in the Engadine. Ischl, in my opinion, has superior attractions for "after-cure" patients. The climate is equable, the diet good, the hotels comfortable, and the adjacent country interesting. For instance, one is then close to the mines of Salzburg; the Austrians frequent Ischl; variety and diversion in the surrounding life are thus obtainable. St. Moritz is situated at an elevation six thousand feet above the sea. It is a beautiful valley, surrounded by imposing scenery. But the climate is variable; they have but one really comfortable month, and that is sometimes July and sometimes August. The adage of the inhabitants is "nine months winter and three months cold." I was there in the latter part of July and the first part of August; variations of temperature were frequent, as much as fifteen or twenty degrees sometimes in a day, the average height of the thermometer being 60° Fahr. to 65° Fahr. The sun at mid-day is often hot, but the climate is too cold for anæmic people. The hotels are not well kept, and, what is worse, the drainage of both hotels and village is conducted into the river Inn. This river runs low during August; the drainage pipes are thus exposed above the surface of the water, and the air around the large hotels is very impure. Were it not for the elevation and the wonderfullly exhilarating alpine air, I fear the effect of this vitiated atmosphere would be very noticeable. The matter of drainage is equally imperfect at Carlsbad, and it is a serious drawback to its otherwise great advantages.

I can recommend, however, the waters of St. Moritz. The proportion of iron is very small, the water is universally well digested, and, as it is rendered sparkling by the carbonic acid, it is very acceptable to the taste. The baths, which are a feature of the place, are very agreeable. Water from a spring similar to that used for drinking is employed, and the stimulating effect of the carbonic acid to the skin reminds one of the exhilarating effect of an ocean-bath. The waters are warmed by steam to any desired temperature, and after leaving a bath the circulation is equalized, and, as I said, the sensation is one of exhilaration. The bath rooms are comfortable, but the tubs are wooden, without tile lining, so common elsewhere, and I fear they are not as clean as might be.

The remarks on the climatology of St. Moritz do not apply to the other regions in the Engadine. Pontresina, a village a few miles distant, is situated in a smaller valley, and is much less visited by high winds; but, as there is no iron spring, a daily ride to St. Moritz is obligatory. After a course of life as above described, careful diet is most important, and, although it is said to be well to leave these high latitudes gradually, after conversing with many travelers I incline to think a return to Paris or England, or for Americans a return to the United States, is far better, even at the cost of returning a second time to Carlsbad. Fish, oysters and fresh vegetables are articles of supreme importance in a dietary, and these cannot be procured unless in the localities I have named.

The above facts are personal observations. I have no personal testimony as to the value of the waters in the treatment for prevention of calculii, renal or hepatic.

For a treatise on the former subject I recommend the perusal of Sir Henry Thompson's work, already alluded to. For the latter, the assertions of J. Kraus must be consulted. To his work I must also refer for a statement that these waters are of value in the treatment of diabetes, although no details are given as to results.

At Carlsbad the Giesshubler Lauerbrunn water is not recommended as a suitable drinking water, the imperfect draining of the place rendering the water undrinkable. It is a very feebly alkaline water, of agreeable taste, but, as an alkaline water, inferior to Vichy or seltzer water, in my estimation.—*Medical Times.*

